# Can centrifugation improve cryotolerance of bovine embryos produced *in vitro*?

**DOI:** 10.1590/1984-3143-AR2024-0123

**Published:** 2025-11-10

**Authors:** Danieli Aparecida Bóbbo Moreski, Josmar Mazucheli, Fabio Luiz Bim Cavalieri, Anthony Cesar de Souza Castilho, Anne Kemmer Souza, Camila Bortoliero Costa, Marcelo Marcondes Seneda, Isabele Picada Emanuelli

**Affiliations:** 1 Universidade Cesumar – Unicesumar, ICETI, Maringá, PR, Brasil; 2 Instituto Cesumar de Ciência Tecnologia e Inovação – ICETI, Maringá, PR, Brasil; 3 Departamento de Estatística, Universidade Estadual de Maringá – UEM, Maringá, PR, Brasil; 4 Universidade do Oeste Paulista – UNOESTE, Presidente Prudente, SP, Brasil; 5 Universidade Estadual de Londrina – UEL, Londrina, PR, Brasil

**Keywords:** blastocyst, bovine, cryopreservation, lipid, oocytes

## Abstract

We tested the effects of centrifuging in vitro matured bovine oocytes for varying times on embryo development and cryotolerance. The oocytes were divided into four groups: control (GC) and centrifuged groups [5433 x g: G5, n = 463 (5 min); G10, n = 461 (10 min); and G15, n = 483 (15 min)]. After centrifugation, the oocytes underwent *in vitro* fertilization for embryo production. Two parameters were evaluated: i) embryonic development (n = 1,878), and ii) cryotolerance evaluation (survival and hatching rates; n = 303). The CG and G10 groups showed blastocyst rates of 42.25% and 45.77%, respectively, higher than those of the other groups (p = 0.02). The hatching rate was equal (p > 0.05) in CG (91.96%), G5: (87.74%), and G10: (95.73%) groups; however, it was lower in G15: 77.06% (p < 0.01). In the CG group, 65.88% of cryopreserved embryos survived, which was different (p < 0.05) from that in G5 (82.02%) and G10 (82.28%) (p > 0.05). Post-freeze hatching percentage was 74.0%, 87.7%, and 47.7%, in G5, G10, and G15, respectively, which was significantly greater than that in CG (p < 0.01; 26.8%). Post-freeze hatching percentage in only G10 matched that of the non-cryopreserved embryos CG (p = 0.06, 92%). We conclude that oocyte centrifugation for 10 minutes was efficient for in vitro embryonic development and cryopreservation of cattle embryos.

## Introduction

Lipids are essential cellular biomolecules that are involved in the initial metabolism of embryos, providing energy for embryonic development (Prates et al., 2014). Excessive accumulation of intracellular lipid droplets may cause cellular damage, resulting in low-quality embryos produced in vitro (Prates et al., 2014). The stressful conditions to which the oocyte may be subjected prior to embryonic development, as well as the conditions of the culture medium during embryo development, are likely the causes of the high lipid content (Sanches et al., 2013; Paschoal et al., 2017). Because of the relationship between lipid content and cryotolerance in bovine, different strategies have been studied (Sudano et al., 2011). Although these strategies often result in a reduced lipid content, they can adversely affect embryonic gene expression and morphology (Annes et al., 2023), and enhanced cryotolerance is not always achieved.

Physical centrifugation has been used to redistribute cytoplasmic lipids in immature bovine and porcine oocytes (Chung et al., 2001; Hara et al., 2005), as well as in early-stage porcine embryos; however, when applied to more advanced embryonic stages, it resulted in lower blastocyst rates (Li et al., 2009). Although Chung et al. (2001) also applied centrifugation to *in vitro* matured bovine oocytes at the MII stage, their study focused on different centrifugation speeds and did not assess cryotolerance. To our knowledge, no previous studies have investigated the impact of centrifugation duration on lipid redistribution and subsequent cryotolerance of bovine oocytes matured *in vitro*. Therefore, in this study, we evaluated three centrifugation time-based protocols for bovine oocytes, aiming to modulate the lipid content of *in vitro* produced embryos and determine the effects on embryo survival after cryopreservation.

## Methods

### Animals

This study was conducted in accordance with the Ethics Committee on Animal Experimentation of the State University of Londrina based on Federal Law 11,794 on October 8, 2008. All animal experiments were performed in accordance with the ARRIVE guidelines, the U.K. Animals (Scientific Procedures) Act of 1986, and associated guidelines (EU Directive 2010/63/EU). The study protocol was approved by the Ethics Committee on Animal Experimentation of the State University of Londrina (CEUA n° 031.2024; OF. CIRC. CEUA n° 061/2024).

Bovine ovaries (n=248) were obtained from the commercial slaughterhouse from females with a predominantly *Bos taurus indicus* phenotype of the Nellore breed. The ovaries were collected, packaged, and transported to the laboratory in 0.9% (w/v) saline solution at 30 and 35 °C. Only follicles with a diameter of 2–8 mm were aspirated with hypodermic needles (30 × 8; 21G) attached to 10 mL syringes for the recovery of *cumulus*–oocyte complexes (COCs). The collection of COCs occurred within 4 hours after the ovaries were obtained from the slaughterhouse.

### In vitro embryo production (IVP) and experimental design

The procedures for in vitro embryo production were conducted according to the protocol described by Costa et al. (2024), with minor modifications. For in vitro maturation (IVM), only COCs classified as quality I or II based on criteria described previously (Seneda et al., 2001) were used. After 24 hours of maturation, the COCs were then randomly divided into four groups (20 oocytes per group): CG - Control group (not centrifuged); G5 - centrifugation at 5433×g for 5 min; G10 centrifugation at 5433×g for 10 min or G15 centrifugation at 5433×g for 15 minutes. All centrifugations were performed using a MiniSpin® centrifuge (Eppendorf, 2020; radius = 6 cm) at 9000 rpm, resulting in a relative centrifugal force (RCF) of 5433 × g. After this step, oocytes were subjected to in vitro fertilization using semen from a single Nelore bull to control for sire-related variability across replicates. The entire experiment was conducted over ten independent replicates involving ovary collection and in vitro embryo production (IVP) procedures.

Two evaluations were then conducted: i) assessment of embryonic development- the oocytes from all groups (n=1878) were subjected to *in vitro fertilization* and *in vitro culture*; ii) evaluation of cryotolerance- total blastocyst (n= 303; quality 1) from the four treatments were cryopreserved using a slow freezing method (Dominium's Programmable Cellular Freezer, BIOCOM®). The methodology used was based on Sanches et al., 2016, with minor modifications. Post-thaw culture was performed in SOF medium supplemented with 2.5% fetal bovine serum (FBS) and 30 mg/mL fatty acid-free BSA, under the same conditions used for pre-freezing embryo culture. Embryo survival was assessed 12 hours after thawing. Post-thaw blastocysts were then cultured for 72 hours in SOF medium supplemented with 2.5% fetal bovine serum (FBS) and 30 mg/mL fatty acid-free BSA. Hatching rates were evaluated at the end of the 72-hour culture period (Sanches et al., 2016).

Additionally, the non-cryopreserved control group (CG*) consisted of blastocysts from the CG group that were produced within the same ten replicates as the experimental groups. These embryos were not subjected to cryopreservation and remained in culture during the same period. Cryopreserved embryos were frozen and immediately thawed, then cultured in parallel with the CG* group under identical conditions to allow simultaneous evaluation.

The assessment schedules for embryo development and cryotolerance were standardized as follows: cleavage rate was evaluated on Day 3, blastocyst formation on Day 7, and hatching rate on Day 9 of in vitro culture. Cryopreservation was performed on Day 7 for embryos that had reached the blastocyst stage.

### Statistical analysis

The Chi-square test was used to compare the rates of embryonic cleavage, blastocyst production, hatching, embryonic survival, and hatching after thawing. Analyses were performed using the SAS software, version 9.1. Differences were considered significant when *p* ≤ 0.05.

## Results

There was no significant difference in cleavage rates among the groups. However, the proportion of blastocysts was lower in the G5 and G15 groups compared to CG and G10. The hatching rate of 77.0% in the G15 group significantly differed (p < 0.0001) from that of all other groups (Table 1).

**Table 1:** Embryonic development after in vitro fertilization of mature bovine oocytes subjected to lipid translocation using centrifugation at 5433 × g for different times.

**Group**	**Centrifugation**	**Oocyte (n)**	**Cleavage % (n)**	**Blastocyst % (n)**	**Hatching % (n)**
**CG**	-	471	77.07 (363/471) ^a^	42.25 (199/471) ^a^	91.96 (183/199) ^a^
**G5**	5 min	463	73.65 (341/463) ^a^	33.48 (155/463) ^b^	87.74 (136/155) ^a^
**G10**	10 min	461	74.19 (342/461) ^a^	45.77 (211/461) ^a^	95.73 (202/211) ^a^
**G15**	15 min	483	71.22 (344/483) ^a^	35.20 (170/483) ^b^	77.06 (133/170) ^b^

(-): Indicates that the technique specified was not performed in the group. ^a, b^Different letters indicate statistically significant differences (p < 0.05) between groups in the same column.

Considering their morphological quality, the blastocysts from the control group exhibited lower tolerance to cryopreservation compared to those from the other groups. Assessment of blastocyst re-expansion 12 hours after thawing revealed a statistically significant difference between the control group (CG) and both G5 (p=0.0165) and G10 (p=0.0187) (Table 2).

**Table 2:** Evaluation of the 12-h re-expansion and 72-h hatching rates (post-thaw) in bovine blastocysts produced in vitro with bovine oocytes subjected to lipid translocation by centrifugation at 5433 × g.

**Group**	**Centrifugation**	**Blastocyst**	**Cryopreservation**	**Reexpansion 12h %**	**Hatching 72 h %**
**CG**	-	50	No (-)	NA	92.00 (46/50) ^a^
**CG+**	-	85	Yes (+)	65.9(56/85) ^a^	26.8 (15/56) ^b^
**G5+**	5 min	89	Yes (+)	82.0 (73/89) ^b,c^	74.3. (54/73) ^c^
**G10+**	10 min	79	Yes (+)	82.3 (65/79) ^b,c^	87.7 (57/65) ^a,c^
**G15+**	15 min	80	Yes (+)	78.8 (63/80) ^a,c^	47.7 (30/63) ^d^

(-) indicates that the specified technique was not performed in the group; (+) fresh, non-cryopreserved group. Laboratory production control. ^a, b^Different letters indicate a statistically significant difference (p < 0.05) between groups in the same column.

The hatching rates of blastocysts after freezing (72 h post-thawing) showed significant differences between CG and the G5, G10, and G15 groups. Notably, only the 10-minute centrifugation group (G10) exhibited a hatching rate similar to the non-cryopreserved control group (CG*; p = 0.0694).

## Discussion

Centrifugation proved useful in overcoming the limitations of cryopreservation of in vitro produced embryos, as it improved post-freezing embryo survival. In the present study, blastocysts derived from centrifuged oocytes showed higher post-thaw re-expansion rates compared to those from the non-centrifuged control group, and also compared favorably with reported rates by Diez et al. (2001) in cattle embryos that underwent centrifugation and micromanipulation-based delipidation at the zygote stage, including a group that received only centrifugation combined with cytochalasin treatment (30–40% re-expansion). Notably, 72 h after thawing, the embryos from the oocyte group centrifuged for 10 min showed similar hatching rates to those of the non-cryopreserved control group — a significant result for developing strategies to enhance embryo cryotolerance.

Given the relevance of improving cryopreservation techniques, previous experiments in porcine embryos used high-speed centrifugation (12,000–16,000 × *g*) to promote lipids displacement (Nagashima et al., 1995; Ushijima et al., 2004).However, subsequent findings suggested that such high centrifugation forces could negatively affect the structural integrity of in vitro matured bovine oocytes (Chung et al., 2001) and porcine immature oocytes (Hara et al., 2005), reinforcing the advantage of using a lower speed (5433 × g), as applied in the present study. While Chung et al. (2001) also applied centrifugation to *in vitro* matured bovine oocytes, their study investigated different centrifugation speeds and did not evaluate embryo development or cryotolerance. In contrast, our work focused on the impact of centrifugation duration and its effects on post-thaw embryo survival, representing a novel contribution to the field.

Additionally, Li et al. (2009) showed that earlier application of centrifugation during porcine embryo development (2–4 cell stages) improved blastocyst rates, but their approach involved cleavage-stage embryos and trypsin treatment, differing from the oocyte-stage protocol we evaluated. Compared with these previous studies, the centrifugation method described here offers advantages in terms of simplicity, cost-effectiveness and most importantly, the generation of cryopreserved embryos with survival and hatching outcomes comparable to those of fresh embryos.

## Conclusion

In conclusion, the present study demonstrated that 10 minutes of oocyte centrifugation was efficient for the improvement of in vitro embryonic development and cryopreservation of cattle embryos.

## Data Availability

Research data is available in the body of the article.
